# Substituted imidazopyridazines are potent and selective inhibitors of *Plasmodium falciparum* calcium-dependent protein kinase 1 (*Pf*CDPK1)

**DOI:** 10.1016/j.bmcl.2013.03.017

**Published:** 2013-05-15

**Authors:** Timothy M. Chapman, Simon A. Osborne, Nathalie Bouloc, Jonathan M. Large, Claire Wallace, Kristian Birchall, Keith H. Ansell, Hayley M. Jones, Debra Taylor, Barbara Clough, Judith L. Green, Anthony A. Holder

**Affiliations:** a Centre for Therapeutics Discovery, MRC Technology, Mill Hill, London NW7 1AD, UK; b Division of Parasitology, MRC National Institute for Medical Research, The Ridgeway, Mill Hill, London NW7 1AA, UK

**Keywords:** *Plasmodium falciparum*, Calcium-dependent protein kinase 1, Malaria, Imidazopyridazine, SAR

## Abstract

A series of imidazopyridazines which are potent inhibitors of *Plasmodium falciparum* calcium-dependent protein kinase 1 (*Pf*CDPK1) was identified from a high-throughput screen against the isolated enzyme. Subsequent exploration of the SAR and optimisation has yielded leading members which show promising in vitro anti-parasite activity along with good in vitro ADME and selectivity against human kinases. Initial in vivo testing has revealed good oral bioavailability in a mouse PK study and modest in vivo efficacy in a *Plasmodium berghei* mouse model of malaria.

## Graphical abstract

**Figure:**
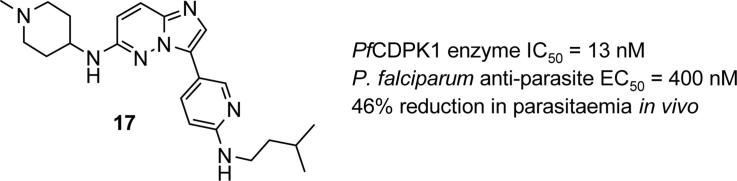

Malaria is one of the most prevalent infectious diseases of the developing world. In excess of 3 billion people are at risk, and it currently leads to the deaths of almost 1 million people each year, with the majority of these occurring in sub-Saharan Africa among children under 5 years of age.^1^ Resistance to existing anti-malarial drugs is widespread^2^ and therefore new therapeutic approaches are urgently needed. Calcium-dependent protein kinases (CDPKs) are directly regulated by Ca^2+^ and are found in plants and organisms in the alveolate lineage,^3^ but are absent in humans. They are present in *Apicomplexan* parasites including *Plasmodium falciparum*, the causative agent of the most severe form of malaria. CDPKs in *Plasmodium* are present as a multigene family containing at least five members,^4^ and different CDPKs are proposed to be functional at different stages of the parasite life cycle. *P. falciparum* calcium-dependent protein kinase 1 (*Pf*CDPK1), first identified by Zhao et al.,^5^ is expressed in the asexual blood stages of the parasite responsible for disease pathology. It has been shown to be encoded by an essential gene^6^ and it is implicated in parasite motility and host cell invasion, where it is able to phosphorylate components of the molecular motor that drive parasite invasion of red blood cells.^7^ If this invasion process can be prevented the parasite lifecycle would be broken, leading the parasites to die and the infection to be cleared. *Pf*CDPK1 therefore represents a novel target for the potential treatment of malaria and offers promise for achieving selectivity over the kinases of the human host. More recently its role in translational regulation of motor complex transcripts has been reported^8^ but hitherto few inhibitors of *Pf*CDPK1 have been described in the literature.^9^

A high throughput screen of our compound collection against the isolated recombinant *Pf*CDPK1 enzyme was performed^10^ and a series containing a 3,6-disubstituted imidazopyridazine core template was identified as the primary series of interest (Fig. 1). Early examples with R^1^ as a 2- or 3-aminoethylpyridyl group and R^2^ as a phenyl ring carrying an appended amide, cyano or fluoro group all showed sub-100 nanomolar IC_50_ values against the enzyme (Table 1). Initial screening of these compounds against the *P. falciparum* parasite in vitro showed strong inhibition of parasite growth in a number of cases. However, despite the promising potency of these early compounds, they typically showed high log *D* values and low stability in microsomes. Furthermore, they exhibited poor selectivity for *Pf*CDPK1 over a panel of human kinases, and their anti-parasite effect may be driven by significant off-target activity. Related imidazopyridazines have been described in the literature as effective inhibitors of other kinases such as human PIM kinase,^11^ IKKβ^12^ and malarial *Pf*PK7.^13^

**Figure 1:**
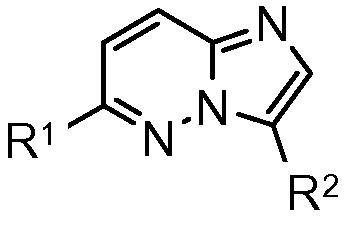
3,6-Imidazopyridazine hit core template.

**Table 1:** Examples from hit series

Compound	R^1^	R^2^	*Pf*CDPK1 IC_50_ (μM)	*P. falciparum* growth inhibition^a^ (%)
**1**			0.059	99
**2**			0.061	99
**3**			0.066	35
**4**			0.071	98
**5**			0.080	6

a At 1 μM inhibitor concentration.

The aim at this point was to explore SAR towards improving the potency alongside the selectivity, ADME and physical properties of the series. In order to assist compound design, a homology model of *Pf*CDPK1 was built based on the published crystal structure of *Tg*CDPK1^14^ and docking studies using Glide (Schrödinger Inc.) suggested that the imidazopyridazine core could form a key H-bond interaction between the nitrogen at the 1-position and the backbone N-H of Tyr-148 at the kinase hinge region (Fig. 2).

**Figure 2:**
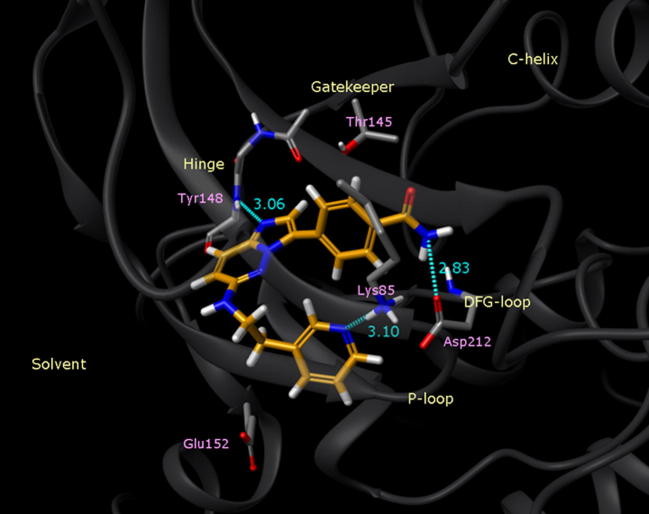
Proposed binding orientation from docking of compound **1** in homology model of *Pf*CDPK1.

The aminoethylpyridine group at R^1^ could form an interaction with the Lys-85 but was also directed towards the Glu-152 residue at the entrance to the pocket, leading out towards solvent. The R^2^ portion was proposed to occupy a pocket where the model suggested there was sufficient space to append larger groups onto the phenyl ring. This offered a potential opportunity to gain improved potency and selectivity in comparison to compounds such as **1** and **2**.

Synthesis of a range of analogues with variation of the groups at both R^1^ and R^2^ was undertaken in order to build the SAR, and examples given in Table 2 illustrate the results from assays against both the *Pf*CDPK1 enzyme and *P. falciparum* parasite.^15^ It was rapidly found that the pyridyl group at the R^1^ position of the molecule was less important in contributing to the binding affinity than the core and R^2^ groups, so this R^1^ could be replaced with a more basic amine group with the aim of lowering the log *D* and improving the ADME and physical properties of the compounds. Exploration of a range of different basic amine side chains at R^1^ revealed that *N*-methyl piperidine and 1,4-diaminocyclohexane in particular gave good enzyme affinity. At the R^2^ position, N-linked phenyl amides and carbamates showed good enzyme affinity and sub-micromolar EC_50_ values against the *P. falciparum* parasite (Table 2, examples **6**–**8**). C-linked phenyl amides also showed good enzyme affinity: a range of different alkyl groups were investigated and the isopentyl group was found to be optimal for enzyme affinity (examples **9** and **10**) with sub-micromolar anti-parasite EC_50_. Compounds were prepared following the synthetic route shown in Scheme 1: installation of the basic amine side chain was achieved by nucleophilic substitution at the 6-chloro substituent of **11** to afford the intermediates **12** and **14**. The 3-position N-linked amides or carbamate **6**–**8** were accessed by Suzuki coupling either directly or through the intermediate aniline **15** with subsequent functionalisation. The 4-position C-linked amides were accessed by Suzuki coupling followed by hydrolysis to give the carboxylic acids **13** and **16** then amide coupling with isopentyl amine.

**Table 2:** SAR with basic amine groups at R^1^ and substituted phenyl groups at R^2^

Compound	R^1^	R^2^	*Pf*CDPK1 IC_50_ (μM)	*P. falciparum* EC_50_ (μM)
**6**			0.011	0.32
**7**			0.022	0.56
**8**			0.018	0.75
**9**			0.016	0.46
**10**			0.023	0.78

**Scheme 1:**
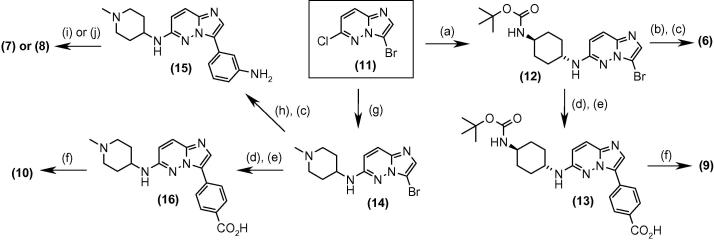
Reagents and conditions: (a) 1,4-cyclohexanediamine, dioxane/NMP, microwave, 180 °C then di-*tert*-butyldicarbonate, DMAP, CH_3_CN, 50 °C; (b) 3-acetamidophenylboronic acid pinacol ester, Pd(dppf)Cl_2_, aq Na_2_CO_3_, dioxane, reflux; (c) 4 M HCl/dioxane; (d) 4-ethoxycarbonylphenylboronic acid, Pd(dppf)Cl_2_, aq Na_2_CO_3_, dioxane, reflux; (e) LiOH, THF/MeOH/H_2_O; (f) isopentylamine, TBTU, DIPEA, and DMF; (g) 1-methyl-4-aminopiperidine, NMP, microwave, 180 °C; (h) 3-*N*-*tert*-butoxycarbonylaminophenylboronic acid, Pd(dppf)Cl_2_, aq Na_2_CO_3_, dioxane, reflux; (i) cyclopropanecarbonyl chloride, DIPEA, and CH_2_Cl_2_; (j) methyl chloroformate, DIPEA, CH_2_Cl_2_.

In order to try to further improve the physical properties of the compounds, decrease the log *D* and improve anti-parasite potency, replacement of the phenyl ring attached to the imidazopyridazine core with a heteroaryl ring was investigated.

The replacement of the phenyl ring by pyridyl and directly linking the alkylamine to the pyridyl ring resulted in a compound with good enzyme affinity and sub-500 nanomolar cell potency (Table 3, example **17**), which also displayed a good in vitro ADME profile (see Table 6). A range of alternative alkyl groups was explored and while changes could be accommodated (e.g., **18** and **19**), none were superior to the isopentyl group for potency. The introduction of polarity led to a small loss in potency (**20**) and the alternative pyridine isomer carrying the isopentylamine substituent (**21**) displayed a sevenfold loss in potency against the enzyme in comparison to **17**. The compounds were obtained through the synthetic route shown in Scheme 2: Suzuki coupling gave the chloropyridine intermediates **22** and **23** and the alkylamines were subsequently introduced by nucleophilic displacement.

**Table 3:** SAR with heteroaryl R^2^ (nt = not tested)

**Figure:**
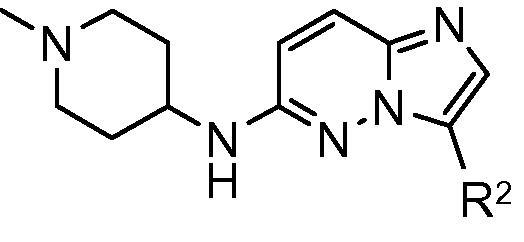

Compound	R^2^	*Pf*CDPK1 IC_50_ (μM)	*P. falciparum* EC_50_ (μM)
**17**		0.013	0.40
**18**		0.014	0.43
**19**		0.036	0.45
**20**		0.025	0.67
**21**		0.088	nt

**Table 4:** SAR with alternative basic amine groups

**Figure:**
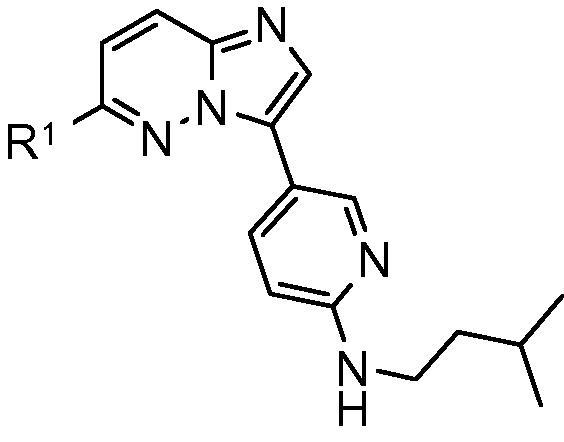

Compound	R^1^	*Pf*CDPK1 IC_50_ (μM)	*P. falciparum* EC_50_ (μM)
**24**		0.023	0.17
**25**		0.089	0.93
**26**		0.175	2.40
**27**		0.044	0.57
**28**		0.013	0.14
**29**		0.015	0.24

**Table 5:** SAR profiles with alternative R^2^ heteroaryl groups or heteroatom linkers (nt = not tested)

**Figure:**
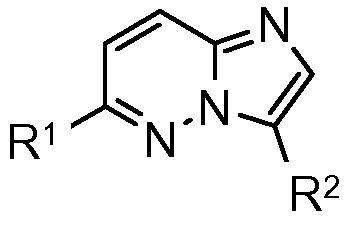

Compound	R^1^	R^2^	*Pf*CDPK1 IC_50_ (μM)	*P. falciparum* EC_50_ (μM)
**30**			0.170	0.54
**31**			0.014	0.47
**32**			0.016	0.17
**33**			0.424	nt

**Table 6:** In vitro ADME and in vivo efficacy data for selected compounds

	Compound			
	**17**	**24**	**27**	**28**
*Pf*CDPK1 IC_50_ (μM)	0.013	0.023	0.044	0.013
*P. falciparum* EC_50_ (μM)	0.40	0.17	0.57	0.14
MLM^a^ (% rem)	63	74	84	90
HLM^a^ (% rem)	85	63	80	90
*m* log *D*	3.4	3.5	3.2	2.5
PAMPA P_app_ (nm s^−1^)	81	114	137	55
Reduction in parasitaemia in vivo^b^ (%)	46	30	7	11

a % Remaining at 30 min.

b 4-Day Peters test in *P. berghei* mouse model, with oral dosing once daily at 50 mg/kg; compounds were dissolved or suspended in 70/30 Tween-80/ethanol and diluted 10-fold with water before dosing.

**Scheme 2:**
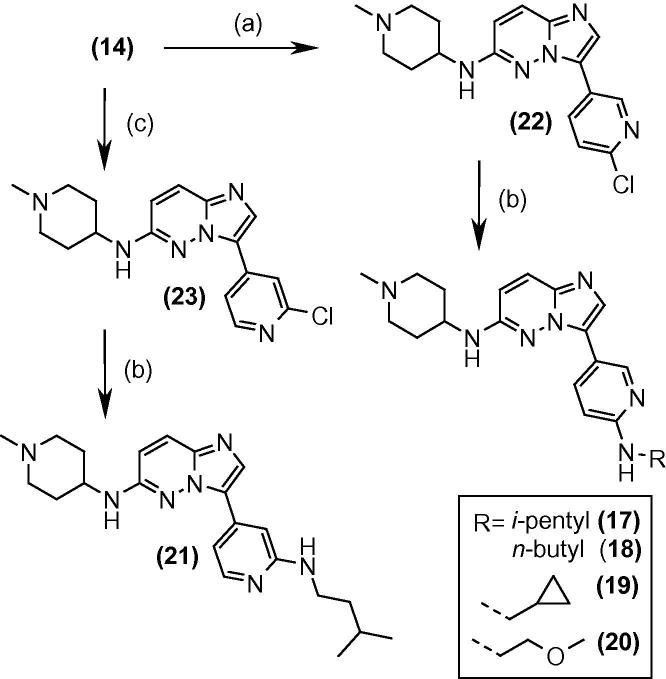
Reagents and conditions: (a) 2-chloro-5-pyridine boronic acid, Pd(dppf)Cl_2_, aq Cs_2_CO_3_, THF, reflux; (b) RNH_2_, NMP, microwave, 190 °C; (c) 2-chloro-4-pyridine boronic acid, Pd(dppf)Cl_2_, aq Cs_2_CO_3_, THF, reflux.

Variation in the basic side-chain at R^1^ with constant R^2^ was then explored (Table 4). This showed that reducing the size of the ring to the pyrrolidine was well tolerated (**24**), however the azetidine (**25**) lost significant potency against both the enzyme and parasite, and this was also observed for the *N*-methyl piperazine (**26**). As predicted by the homology model the presence of the NH was not found to be essential: although the 4-dimethylaminopiperidine (**27**) was less potent than compound **17**, its desmethyl analogue (**28**) showed good potency against both the enzyme and parasite. Similarly, the compound **17** analogue without the *N*-methyl group (**29**) was well accommodated, with no loss in enzyme binding affinity and slightly improved anti-parasite activity.

Returning to the R^2^ position, further changes in the heteroaryl ring and the appended groups were investigated (Table 5). The nitrogen linker atom between the heteroaryl ring and the alkyl chain was replaced with an oxygen atom, by performing a nucleophilic substitution with isopentyl alcohol deprotonated with sodium hydride in place of the amine. However, the product (**30**) showed a significant loss in potency, indicating the importance of this N–H donor. Replacement of the pyridine ring with pyrimidine was investigated, and this revealed that compounds containing the pyrimidine attached to the core through the 5-position (**31** and **32**) showed good inhibitory activity whereas attachment at the 4-position (**33**) resulted in a significant loss of potency against the enzyme.

The synthetic routes used to access these analogues are detailed in Scheme 3: installation of the *S*-methyl pyrimidine through Suzuki coupling on the BOC-protected compounds **34** and **12** gave the intermediates **35**–**37**. These were functionalised through oxidation of the *S*-methyl group using mCPBA and subsequent introduction of the alkylamine by nucleophilic substitution. The sequence was completed by removal of the BOC protecting group and then in the case of the piperidine by the introduction of the *N*-methyl group through reductive amination.

**Scheme 3:**
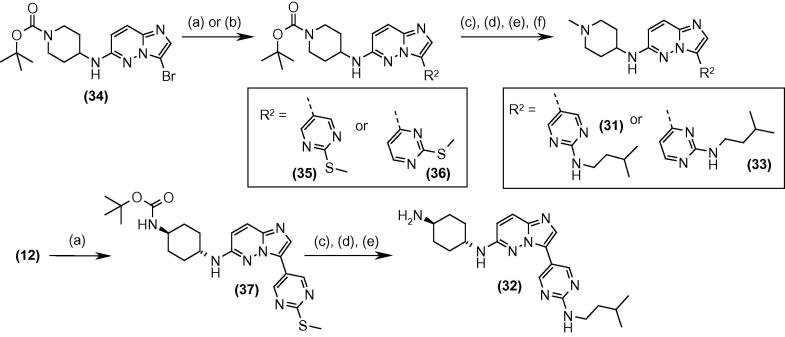
Reagents and conditions: (a) 2-(methylthio)pyrimidine-5-boronic acid pinacol ester, Pd(dppf)Cl_2_, aq Cs_2_CO_3_, dioxane, reflux; (b) 2-(methylthio)pyrimidine-4-boronic acid pinacol ester, Pd(dppf)Cl_2_, aq Cs_2_CO_3_, dioxane, reflux; (c) *m*-chloroperoxybenzoic acid, CH_2_Cl_2_; (d) isopentylamine, dioxane, 65 °C; (e) 4 M HCl/dioxane; (f) formaldehyde, AcOH, Na(OAc)_3_BH, THF.

Overall, the introduction of a basic side chain at the R^1^ position and a heteroaryl ring with an appended aminoalkyl group at R^2^ led to improved potency, physical properties and in vitro ADME characteristics compared with the initial hits. These compounds displayed lower log *D* and higher stability in both mouse and human microsomes alongside significant improvements in kinase selectivity against a human kinase panel.

Compounds possessing the best profiles with respect to potency, in vitro ADME and selectivity were advanced to testing for in vivo efficacy in a *P. berghei* mouse model of malaria. In advance of in vivo testing, it was shown that the inhibitors retained potency against the isolated *P. berghei* CDPK1 enzyme.^16^ Compounds were dosed with an oral, once daily 50 mg/kg regime over 4 days in the standard Peters test, and their in vitro ADME and in vivo efficacy data is shown in Table 6. The best efficacy was displayed by compound **17**, with a 46% reduction in the level of parasitaemia relative to vehicle. This offers promise at this stage considering the relatively modest cellular potency of these compounds and **17** represents an interesting early lead. PK profiling of compound **17** revealed that it possessed a half-life of 2 h and good oral bioavailability in mouse (Fig. 4), although it displayed moderate to high clearance.

**Figure 3:**
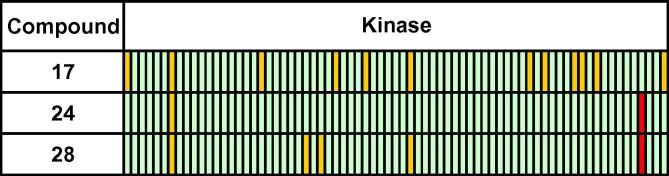
Kinase selectivity data on selected compounds screened at 1 μM inhibitor concentration against a 73-member human kinase panel; green: <50% inhibition; amber: 50–80% inhibition; red: >80% inhibition. Kinases hit by compound **17** are: MKK1, RSK1, PKD1, CHK2, Aurora B, NUAK1, GCK, MLK1, Src, Lck, YES1, and VEGFR; compound **24**: RSK1 and HER4; compound **28**: RSK1, CAMK1, PHK, NUAK1, and HER4.

**Figure 4:**
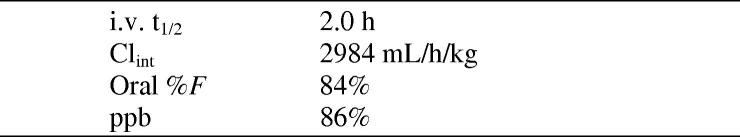
Mouse pharmacokinetic and plasma-protein binding data for compound **17**.

Compounds **17**, **24** and **28** exhibited good selectivity profiles when screened against a panel of human kinases at 1 μM inhibitor concentration (Fig. 3).^17^ Pleasingly, screening against the isolated CDPK1 enzyme of the related malarial parasite *Plasmodium vivax* revealed that these compounds were highly potent against this species,^16^ which is also an important human pathogen causing considerable morbidity.

In summary, a series of imidazopyridazines which are potent inhibitors of *Pf*CDPK1 has been identified. Leading compounds have shown promising in vitro anti-parasite activity, in vitro ADME and kinase selectivity profiles and in vivo pharmacokinetics in mouse, with modest in vivo efficacy in a *P. berghei* mouse model of malaria. Improving the in vitro anti-parasite activity, in vivo efficacy and PK profile of this series is the subject of further work and will be described in a future publication.
